# 9-[3-(9*H*-Carbazol-9-yl)-5-iodo­phen­yl]-9*H*-carbazole

**DOI:** 10.1107/S2414314621004284

**Published:** 2021-04-27

**Authors:** Dan Yan

**Affiliations:** a Testing Center, Fuzhou University, Fuzhou 350108, Fujian, People’s Republic of China; University of Aberdeen, Scotland

**Keywords:** crystal structure, carbazole derivative, π–π stacking, supra­molecular network

## Abstract

In the title compound, C_30_H_19_IN_2_, two independent mol­ecules (*A* and *B*) are present in the asymmetric unit, with different conformations. The dihedral angle between the mean planes of the carbazole systems for mol­ecule *A* is 49.1 (2)° compared to 84.0 (1)° for mol­ecule *B*. In the crystal, numerous aromatic π–π stacking inter­actions [shortest centroid–centroid separation = 3.7069 (19) Å] help to establish the three-dimensional supra­molecular network.

## Abstract 2

In the title compound, two independent mol­ecules (*A* and *B*) are present in the asymmetric unit, with different conformations. The dihedral angle between the mean planes of the carbazole systems for mol­ecule *A* is 49.1 (2)° compared to 84.0 (1)° for mol­ecule *B*. In the crystal, numerous aromatic π–π stacking inter­actions [shortest centroid–centroid separation = 3.7069 (19) Å] help to establish the three-dimensional supra­molecular network.

## Graphical abstract

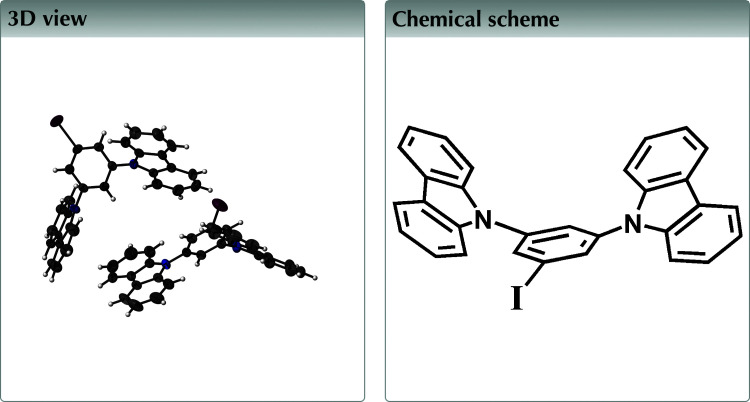

## Structure description

**Figure 1:**
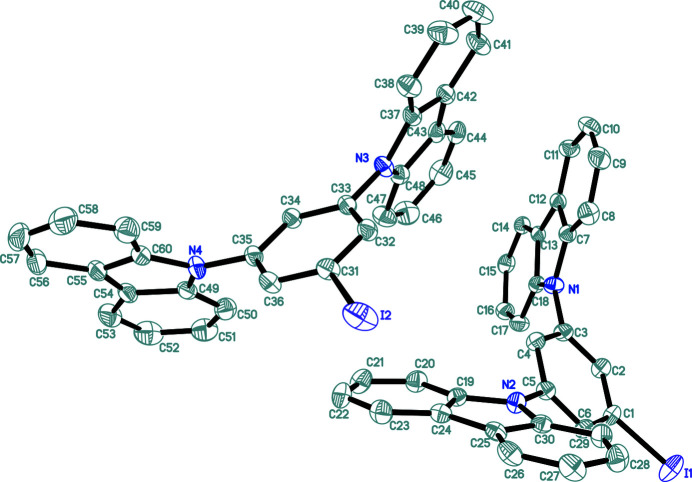
The mol­ecular structure of the title compound showing 30% displacement ellipsoids with H atoms omitted for clarity.

**Figure 2:**
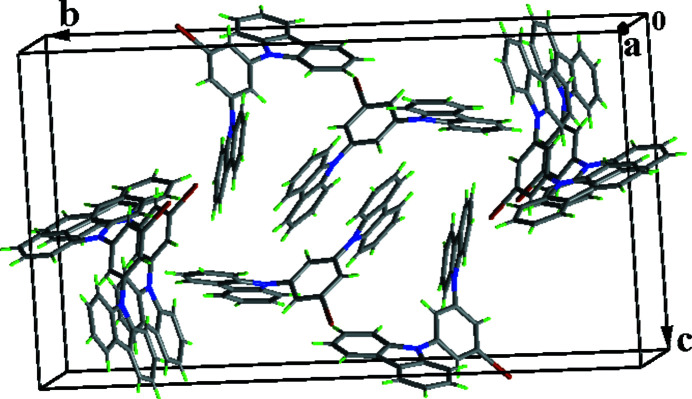
A view of the crystal packing.

Carbazole derivatives display fluorescence and phospho­rescence at room temperature due to their π–π stacking inter­actions (Xie *et al.*, 2017 ▸). The crystal structure of such a compound with mol­ecular formula of C_30_H_19_IN_2_ is reported here. Two independent mol­ecules (*A* and *B*) are present in the asymmetric unit, with different conformations. Each of the independent mol­ecules is composed of two carbazole systems connected by an iodo­benzene bridge (Fig. 1 ▸). The dihedral angle between the mean planes of the N1 and N2 carbazole systems for mol­ecule *A* is 49.1 (2)° compared to 84.0 (1)° for the N3 and N4 ring systems in mol­ecule *B*. In the crystal, numerous face-to-face [shortest centroid–centroid separation = 3.7069 (19) Å] and edge-to-face π–π stacking inter­actions connect the mol­ecules into a three-dimensional supra­molecular network (Fig. 2 ▸).

## Synthesis and crystallization

A mixture of 9*H*-carbazole (3.34 g, 20.0 mmol), *t*-BuOK (2.24 g, 20.0 mmol), and dimethyl sulfoxide (10 ml) was stirred at 120°C before 1-iodo-3,5-di­fluoro­benzene (2.40 g, 10.0 mmol) was injected. The reaction mixture was stirred at 140°C for 2 h. After being cooled, the mixture was extracted with chloro­form and the organic extracts were combined, washed with water, and the organic layer was dried over anhydrous MgSO_4_. After evaporating the solvent, the crude product was purified by column chromatography on silica gel with chloro­form/n-hexane as the eluent to give a white powder. Yield: 83%. ^1^H NMR (500 MHz, chloro­form-*d*) δ 8.17 (*dt*, *J* = 7.8, 1.0 Hz, 4H), 8.08 (*d*, *J* = 1.9 Hz, 2H), 7.84 (*t*, *J* = 1.9 Hz, 1H), 7.56 (*dt*, *J* = 8.2, 0.9 Hz, 4H), 7.49 (*ddd*, *J* = 8.2, 7.1, 1.2 Hz, 4H), 7.35 (*td*, *J* = 7.5, 1.0 Hz, 4H). Colourless blocks were obtained by recrystallization from mixed solvents of methyl­ene chloride and *n*-hexane (*v*:*v* = 1.2).

## Refinement

Crystal data, data collection and structure refinement details are summarized in Table 1 ▸.

## Supplementary Material

Crystal structure: contains datablock(s) I. DOI: 10.1107/S2414314621004284/hb4376sup1.cif

Structure factors: contains datablock(s) I. DOI: 10.1107/S2414314621004284/hb4376Isup2.hkl

Supporting information file. DOI: 10.1107/S2414314621004284/hb4376Isup3.cml

CCDC reference: 2079137

Additional supporting information:  crystallographic information; 3D view; checkCIF report

## Figures and Tables

**Table 1:** Experimental details

Crystal data	
Chemical formula	C_30_H_19_IN_2_
*M*_r_	534.37
Crystal system, space group	Monoclinic, *P*2_1_/*n*
Temperature (K)	296
*a*, *b*, *c* (Å)	9.5057 (4), 29.3757 (17), 16.5679 (8)
β (°)	96.127 (2)
*V* (Å^3^)	4599.9 (4)
*Z*	8
Radiation type	Mo *K*α
μ (mm^−1^)	1.41
Crystal size (mm)	0.25 × 0.23 × 0.23

Data collection	
Diffractometer	Bruker *SMART* *APEX* CCD
Absorption correction	Multi-scan (*SADABS*; Bruker, 1997 ▸)
*T*_min_, *T*_max_	0.719, 0.737
No. of measured, independent and observed [*I* > 2σ(*I*)] reflections	27536, 8124, 6636
*R*_int_	0.030
(sin θ/λ)_max_ (Å^−1^)	0.596

Refinement	
*R*[*F* ^2^ > 2σ(*F* ^2^)], *wR*(*F* ^2^), *S*	0.039, 0.088, 1.04
No. of reflections	8124
No. of parameters	595
H-atom treatment	H-atom parameters constrained
Δρ_max_, Δρ_min_ (e Å^−3^)	0.78, −0.98

Computer programs: *SMART* and *SAINT* (Bruker, 1997 ▸) and *SHELXS97*, *SHELXL97* (Sheldrick, 2008 ▸) and *SHELXTL-Plus* (Sheldrick, 2008 ▸).

## full crystallographic data

### Crystal data

**Table:**

C_30_H_19_IN_2_	*F*(000) = 2128
*M**_r_* = 534.37	*D*_x_ = 1.543 Mg m^−^^3^
Monoclinic, *P*2_1_/*n*	Mo *K*α radiation, λ = 0.71073 Å
*a* = 9.5057 (4) Å	Cell parameters from 296(2) reflections
*b* = 29.3757 (17) Å	θ = 2.3–25.0°
*c* = 16.5679 (8) Å	µ = 1.41 mm^−^^1^
β = 96.127 (2)°	*T* = 296 K
*V* = 4599.9 (4) Å^3^	Block, colourless
*Z* = 8	0.25 × 0.23 × 0.23 mm

### Data collection

**Table:**

Bruker SMART APEX CCD diffractometer	8124 independent reflections
Radiation source: fine-focus sealed tube	6636 reflections with *I* > 2σ(*I*)
Graphite monochromator	*R*_int_ = 0.030
ω scan	θ_max_ = 25.0°, θ_min_ = 2.3°
Absorption correction: multi-scan (SADABS; Bruker, 1997)	*h* = −11→11
*T*_min_ = 0.719, *T*_max_ = 0.737	*k* = −34→34
27536 measured reflections	*l* = −19→19

### Refinement

**Table:**

Refinement on *F*^2^	Primary atom site location: structure-invariant direct methods
Least-squares matrix: full	Secondary atom site location: difference Fourier map
*R*[*F*^2^ > 2σ(*F*^2^)] = 0.039	Hydrogen site location: inferred from neighbouring sites
*wR*(*F*^2^) = 0.088	H-atom parameters constrained
*S* = 1.04	*w* = 1/[σ^2^(*F*_o_^2^) + (0.0331*P*)^2^ + 5.8326*P*] where *P* = (*F*_o_^2^ + 2*F*_c_^2^)/3
8124 reflections	(Δ/σ)_max_ = 0.003
595 parameters	Δρ_max_ = 0.78 e Å^−^^3^
0 restraints	Δρ_min_ = −0.98 e Å^−^^3^

### Special details

**Table:**

Geometry. All esds (except the esd in the dihedral angle between two l.s. planes) are estimated using the full covariance matrix. The cell esds are taken into account individually in the estimation of esds in distances, angles and torsion angles; correlations between esds in cell parameters are only used when they are defined by crystal symmetry. An approximate (isotropic) treatment of cell esds is used for estimating esds involving l.s. planes.
Refinement. Refinement of F^2^ against ALL reflections. The weighted R-factor wR and goodness of fit S are based on F^2^, conventional R-factors R are based on F, with F set to zero for negative F^2^. The threshold expression of F^2^ > 2sigma(F^2^) is used only for calculating R-factors(gt) etc. and is not relevant to the choice of reflections for refinement. R-factors based on F^2^ are statistically about twice as large as those based on F, and R- factors based on ALL data will be even larger.

### Fractional atomic coordinates and isotropic or equivalent isotropic displacement parameters (Å^2^)

**Table:**

	*x*	*y*	*z*	*U*_iso_*/*U*_eq_	
C1	0.3030 (4)	0.30347 (11)	0.9652 (2)	0.0357 (8)	
C2	0.2194 (4)	0.30625 (12)	0.8916 (2)	0.0377 (8)	
H2	0.1401	0.2878	0.8809	0.045*	
C3	0.2562 (4)	0.33710 (12)	0.83435 (19)	0.0354 (8)	
C4	0.3751 (4)	0.36416 (12)	0.8498 (2)	0.0364 (8)	
H4	0.4006	0.3842	0.8104	0.044*	
C5	0.4564 (3)	0.36128 (11)	0.9248 (2)	0.0332 (8)	
C6	0.4186 (3)	0.33118 (11)	0.9835 (2)	0.0355 (8)	
H6	0.4706	0.3298	1.0343	0.043*	
C7	0.2157 (4)	0.32577 (11)	0.6837 (2)	0.0353 (8)	
C8	0.3480 (4)	0.31137 (13)	0.6656 (2)	0.0477 (9)	
H8	0.4250	0.3100	0.7051	0.057*	
C9	0.3598 (5)	0.29915 (14)	0.5863 (3)	0.0535 (10)	
H9	0.4471	0.2894	0.5724	0.064*	
C10	0.2464 (4)	0.30083 (14)	0.5265 (2)	0.0517 (10)	
H10	0.2590	0.2924	0.4737	0.062*	
C11	0.1152 (4)	0.31491 (12)	0.5448 (2)	0.0438 (9)	
H11	0.0388	0.3157	0.5047	0.053*	
C12	0.0987 (4)	0.32798 (11)	0.62428 (19)	0.0337 (8)	
C13	−0.0203 (4)	0.34428 (10)	0.66338 (19)	0.0308 (7)	
C14	−0.1601 (4)	0.35443 (11)	0.6368 (2)	0.0369 (8)	
H14	−0.1938	0.3509	0.5823	0.044*	
C15	−0.2492 (4)	0.36993 (12)	0.6918 (2)	0.0413 (9)	
H15	−0.3433	0.3765	0.6744	0.050*	
C16	−0.1982 (4)	0.37567 (12)	0.7729 (2)	0.0439 (9)	
H16	−0.2594	0.3860	0.8091	0.053*	
C17	−0.0594 (4)	0.36652 (12)	0.8015 (2)	0.0417 (9)	
H17	−0.0262	0.3709	0.8558	0.050*	
C18	0.0293 (4)	0.35050 (11)	0.74595 (19)	0.0329 (7)	
C19	0.5865 (3)	0.43534 (11)	0.92762 (19)	0.0328 (7)	
C20	0.4833 (4)	0.46598 (13)	0.8984 (2)	0.0441 (9)	
H20	0.3915	0.4561	0.8825	0.053*	
C21	0.5176 (4)	0.51059 (14)	0.8933 (2)	0.0512 (10)	
H21	0.4480	0.5315	0.8748	0.061*	
C22	0.6547 (4)	0.52566 (13)	0.9149 (2)	0.0504 (10)	
H22	0.6761	0.5563	0.9097	0.060*	
C23	0.7595 (4)	0.49572 (13)	0.9441 (2)	0.0460 (9)	
H23	0.8513	0.5060	0.9586	0.055*	
C24	0.7261 (4)	0.45019 (12)	0.95148 (19)	0.0353 (8)	
C25	0.8049 (3)	0.41074 (11)	0.98263 (19)	0.0341 (8)	
C26	0.9457 (4)	0.40398 (13)	1.0141 (2)	0.0447 (9)	
H26	1.0089	0.4282	1.0185	0.054*	
C27	0.9894 (4)	0.36122 (14)	1.0382 (2)	0.0515 (10)	
H27	1.0825	0.3566	1.0602	0.062*	
C28	0.8964 (4)	0.32481 (14)	1.0304 (2)	0.0509 (10)	
H28	0.9287	0.2960	1.0469	0.061*	
C29	0.7569 (4)	0.33008 (12)	0.9987 (2)	0.0426 (9)	
H29	0.6954	0.3054	0.9928	0.051*	
C30	0.7121 (3)	0.37348 (11)	0.97597 (19)	0.0338 (8)	
C31	0.9320 (4)	0.52604 (12)	0.7581 (2)	0.0379 (8)	
C32	0.8601 (4)	0.49528 (11)	0.7056 (2)	0.0377 (8)	
H32	0.8932	0.4657	0.7011	0.045*	
C33	0.7369 (4)	0.50968 (11)	0.65984 (19)	0.0337 (8)	
C34	0.6863 (4)	0.55340 (11)	0.6678 (2)	0.0343 (8)	
H34	0.6029	0.5625	0.6377	0.041*	
C35	0.7604 (4)	0.58358 (11)	0.7210 (2)	0.0344 (8)	
C36	0.8849 (4)	0.57009 (12)	0.7663 (2)	0.0395 (8)	
H36	0.9354	0.5903	0.8016	0.047*	
C37	0.7209 (4)	0.45443 (11)	0.54364 (19)	0.0338 (8)	
C38	0.8564 (4)	0.45616 (13)	0.5199 (2)	0.0451 (9)	
H38	0.9251	0.4753	0.5457	0.054*	
C39	0.8854 (5)	0.42831 (15)	0.4566 (2)	0.0577 (11)	
H39	0.9750	0.4290	0.4389	0.069*	
C40	0.7834 (5)	0.39940 (16)	0.4188 (3)	0.0638 (13)	
H40	0.8065	0.3807	0.3769	0.077*	
C41	0.6489 (4)	0.39783 (14)	0.4420 (2)	0.0518 (10)	
H41	0.5813	0.3784	0.4161	0.062*	
C42	0.6154 (4)	0.42606 (11)	0.5053 (2)	0.0360 (8)	
C43	0.4876 (4)	0.43387 (11)	0.5432 (2)	0.0361 (8)	
C44	0.3517 (4)	0.41591 (12)	0.5316 (2)	0.0441 (9)	
H44	0.3286	0.3942	0.4915	0.053*	
C45	0.2528 (4)	0.43036 (15)	0.5793 (3)	0.0569 (11)	
H45	0.1617	0.4185	0.5717	0.068*	
C46	0.2868 (5)	0.46268 (15)	0.6394 (3)	0.0600 (11)	
H46	0.2175	0.4721	0.6713	0.072*	
C47	0.4207 (4)	0.48124 (14)	0.6530 (2)	0.0501 (10)	
H47	0.4430	0.5027	0.6936	0.060*	
C48	0.5203 (4)	0.46660 (11)	0.6036 (2)	0.0351 (8)	
C49	0.5797 (4)	0.64261 (12)	0.7489 (2)	0.0372 (8)	
C50	0.4706 (4)	0.61640 (14)	0.7728 (2)	0.0479 (9)	
H50	0.4767	0.5848	0.7741	0.057*	
C51	0.3520 (5)	0.63869 (16)	0.7946 (3)	0.0589 (11)	
H51	0.2773	0.6218	0.8109	0.071*	
C52	0.3425 (5)	0.68580 (18)	0.7925 (3)	0.0673 (13)	
H52	0.2621	0.7000	0.8077	0.081*	
C53	0.4507 (5)	0.71163 (15)	0.7682 (3)	0.0585 (12)	
H53	0.4435	0.7432	0.7668	0.070*	
C54	0.5713 (4)	0.69033 (12)	0.7456 (2)	0.0424 (9)	
C55	0.7041 (4)	0.70643 (12)	0.7205 (2)	0.0439 (9)	
C56	0.7564 (6)	0.74945 (13)	0.7061 (3)	0.0603 (12)	
H56	0.7023	0.7753	0.7128	0.072*	
C57	0.8889 (6)	0.75312 (16)	0.6819 (3)	0.0731 (15)	
H57	0.9240	0.7818	0.6712	0.088*	
C58	0.9727 (5)	0.71487 (18)	0.6729 (3)	0.0677 (13)	
H58	1.0634	0.7185	0.6576	0.081*	
C59	0.9224 (5)	0.67159 (15)	0.6865 (2)	0.0552 (10)	
H59	0.9769	0.6459	0.6796	0.066*	
C60	0.7879 (4)	0.66813 (12)	0.7107 (2)	0.0397 (8)	
I1	0.25530 (3)	0.255028 (10)	1.050844 (18)	0.05953 (10)	
I2	1.11186 (3)	0.504351 (11)	0.831782 (18)	0.06639 (11)	
N1	0.1724 (3)	0.33975 (10)	0.75786 (16)	0.0364 (7)	
N2	0.5786 (3)	0.38871 (9)	0.94166 (17)	0.0349 (6)	
N3	0.6627 (3)	0.47903 (9)	0.60422 (16)	0.0359 (7)	
N4	0.7111 (3)	0.62920 (9)	0.72709 (18)	0.0387 (7)	

### Atomic displacement parameters (Å^2^)

**Table:**

	*U*^11^	*U*^22^	*U*^33^	*U*^12^	*U*^13^	*U*^23^
C1	0.0306 (19)	0.0364 (19)	0.0402 (19)	0.0020 (15)	0.0044 (15)	0.0111 (15)
C2	0.0299 (19)	0.041 (2)	0.0405 (19)	−0.0051 (16)	−0.0025 (15)	0.0032 (16)
C3	0.0311 (18)	0.041 (2)	0.0318 (17)	0.0011 (16)	−0.0065 (14)	−0.0006 (15)
C4	0.037 (2)	0.0379 (19)	0.0339 (18)	−0.0025 (16)	0.0014 (15)	0.0036 (15)
C5	0.0272 (18)	0.0363 (19)	0.0351 (18)	−0.0006 (15)	−0.0019 (14)	−0.0021 (14)
C6	0.0283 (18)	0.043 (2)	0.0337 (18)	−0.0001 (15)	−0.0047 (14)	0.0050 (15)
C7	0.037 (2)	0.0332 (18)	0.0357 (18)	−0.0018 (15)	0.0020 (15)	0.0002 (14)
C8	0.037 (2)	0.050 (2)	0.055 (2)	0.0056 (18)	−0.0007 (18)	0.0003 (18)
C9	0.049 (3)	0.054 (2)	0.060 (3)	0.011 (2)	0.017 (2)	−0.005 (2)
C10	0.058 (3)	0.053 (2)	0.045 (2)	0.001 (2)	0.014 (2)	−0.0149 (19)
C11	0.048 (2)	0.049 (2)	0.0335 (19)	−0.0040 (18)	−0.0010 (17)	−0.0045 (16)
C12	0.0361 (19)	0.0297 (17)	0.0342 (18)	−0.0021 (15)	−0.0006 (15)	−0.0014 (14)
C13	0.0371 (19)	0.0250 (16)	0.0291 (16)	−0.0031 (14)	−0.0014 (14)	−0.0020 (13)
C14	0.039 (2)	0.0333 (18)	0.0352 (18)	0.0008 (16)	−0.0092 (16)	0.0016 (15)
C15	0.034 (2)	0.042 (2)	0.047 (2)	0.0066 (16)	−0.0041 (17)	0.0064 (17)
C16	0.047 (2)	0.045 (2)	0.040 (2)	0.0063 (18)	0.0074 (17)	−0.0005 (17)
C17	0.046 (2)	0.048 (2)	0.0313 (18)	0.0015 (18)	0.0013 (16)	−0.0034 (16)
C18	0.0337 (19)	0.0303 (17)	0.0332 (18)	−0.0029 (15)	−0.0035 (14)	0.0029 (14)
C19	0.0303 (18)	0.0366 (19)	0.0311 (17)	−0.0018 (15)	0.0016 (14)	0.0007 (14)
C20	0.039 (2)	0.047 (2)	0.044 (2)	0.0016 (18)	−0.0062 (16)	0.0031 (17)
C21	0.047 (2)	0.049 (2)	0.056 (2)	0.0006 (19)	−0.0011 (19)	0.0136 (19)
C22	0.057 (3)	0.036 (2)	0.059 (2)	−0.0054 (19)	0.009 (2)	0.0081 (18)
C23	0.041 (2)	0.047 (2)	0.051 (2)	−0.0162 (18)	0.0053 (18)	−0.0042 (18)
C24	0.0320 (19)	0.041 (2)	0.0330 (18)	−0.0032 (16)	0.0047 (15)	−0.0036 (15)
C25	0.0291 (18)	0.0397 (19)	0.0331 (18)	−0.0022 (15)	0.0025 (14)	−0.0091 (15)
C26	0.0311 (19)	0.049 (2)	0.053 (2)	−0.0046 (17)	−0.0019 (17)	−0.0168 (18)
C27	0.032 (2)	0.060 (3)	0.059 (2)	0.0094 (19)	−0.0087 (18)	−0.014 (2)
C28	0.042 (2)	0.046 (2)	0.064 (3)	0.0111 (19)	−0.0024 (19)	0.0010 (19)
C29	0.038 (2)	0.039 (2)	0.049 (2)	−0.0008 (17)	−0.0030 (17)	0.0001 (17)
C30	0.0293 (18)	0.041 (2)	0.0300 (17)	0.0010 (15)	−0.0005 (14)	−0.0046 (14)
C31	0.0313 (19)	0.045 (2)	0.0359 (18)	0.0098 (16)	−0.0033 (15)	−0.0034 (16)
C32	0.041 (2)	0.0325 (18)	0.0393 (19)	0.0104 (16)	0.0004 (16)	−0.0026 (15)
C33	0.0370 (19)	0.0305 (18)	0.0333 (17)	0.0005 (15)	0.0025 (15)	−0.0028 (14)
C34	0.0343 (19)	0.0325 (18)	0.0353 (18)	0.0055 (15)	−0.0006 (15)	−0.0012 (14)
C35	0.037 (2)	0.0288 (17)	0.0372 (18)	0.0052 (15)	0.0050 (15)	−0.0027 (14)
C36	0.041 (2)	0.038 (2)	0.0392 (19)	0.0017 (17)	0.0008 (16)	−0.0110 (16)
C37	0.035 (2)	0.0308 (18)	0.0341 (18)	0.0088 (15)	−0.0027 (15)	−0.0019 (14)
C38	0.040 (2)	0.051 (2)	0.043 (2)	0.0032 (18)	0.0002 (17)	−0.0061 (17)
C39	0.045 (2)	0.079 (3)	0.050 (2)	0.018 (2)	0.008 (2)	−0.008 (2)
C40	0.060 (3)	0.083 (3)	0.046 (2)	0.027 (3)	−0.004 (2)	−0.024 (2)
C41	0.054 (3)	0.053 (2)	0.044 (2)	0.016 (2)	−0.0134 (19)	−0.0175 (18)
C42	0.040 (2)	0.0324 (18)	0.0335 (18)	0.0088 (16)	−0.0053 (15)	−0.0007 (14)
C43	0.037 (2)	0.0295 (18)	0.0390 (19)	0.0020 (15)	−0.0068 (15)	0.0037 (15)
C44	0.046 (2)	0.035 (2)	0.048 (2)	−0.0024 (17)	−0.0076 (18)	0.0064 (16)
C45	0.039 (2)	0.061 (3)	0.070 (3)	−0.009 (2)	0.003 (2)	0.009 (2)
C46	0.047 (3)	0.072 (3)	0.064 (3)	0.002 (2)	0.020 (2)	−0.001 (2)
C47	0.045 (2)	0.059 (2)	0.048 (2)	−0.005 (2)	0.0119 (18)	−0.0120 (19)
C48	0.0336 (19)	0.0356 (19)	0.0351 (18)	0.0023 (15)	−0.0006 (15)	0.0025 (15)
C49	0.044 (2)	0.0368 (19)	0.0302 (17)	0.0061 (17)	0.0013 (15)	−0.0052 (15)
C50	0.049 (2)	0.046 (2)	0.048 (2)	0.0064 (19)	0.0062 (18)	0.0021 (18)
C51	0.050 (3)	0.074 (3)	0.053 (3)	0.007 (2)	0.008 (2)	0.000 (2)
C52	0.058 (3)	0.083 (4)	0.061 (3)	0.033 (3)	0.007 (2)	−0.006 (2)
C53	0.074 (3)	0.045 (2)	0.055 (3)	0.029 (2)	0.000 (2)	−0.0056 (19)
C54	0.058 (2)	0.0323 (19)	0.0357 (19)	0.0090 (18)	−0.0021 (17)	−0.0060 (15)
C55	0.063 (3)	0.0319 (19)	0.0348 (19)	−0.0008 (18)	−0.0060 (18)	−0.0047 (15)
C56	0.088 (4)	0.034 (2)	0.055 (3)	−0.007 (2)	−0.009 (2)	−0.0021 (18)
C57	0.105 (4)	0.052 (3)	0.057 (3)	−0.036 (3)	−0.015 (3)	0.008 (2)
C58	0.065 (3)	0.079 (3)	0.058 (3)	−0.029 (3)	0.001 (2)	0.005 (2)
C59	0.052 (3)	0.057 (3)	0.057 (3)	−0.008 (2)	0.008 (2)	−0.005 (2)
C60	0.051 (2)	0.0346 (19)	0.0326 (18)	−0.0030 (17)	0.0012 (16)	−0.0043 (15)
I1	0.04082 (16)	0.0682 (2)	0.06848 (19)	−0.00388 (13)	0.00070 (13)	0.03549 (15)
I2	0.04937 (18)	0.0874 (2)	0.05720 (18)	0.03071 (15)	−0.01822 (13)	−0.01873 (15)
N1	0.0299 (16)	0.0466 (17)	0.0309 (15)	0.0000 (13)	−0.0049 (12)	−0.0020 (13)
N2	0.0271 (15)	0.0352 (16)	0.0404 (16)	−0.0023 (12)	−0.0052 (12)	0.0023 (12)
N3	0.0360 (16)	0.0330 (15)	0.0376 (15)	0.0012 (13)	−0.0016 (13)	−0.0087 (12)
N4	0.0417 (18)	0.0266 (15)	0.0483 (17)	0.0016 (13)	0.0068 (14)	−0.0051 (13)

### Geometric parameters (Å, º)

**Table:**

C1—C6	1.375 (5)	C31—C36	1.381 (5)
C1—C2	1.385 (5)	C31—C32	1.383 (5)
C1—I1	2.093 (3)	C31—I2	2.091 (3)
C2—C3	1.383 (5)	C32—C33	1.391 (5)
C2—H2	0.9300	C32—H32	0.9300
C3—C4	1.383 (5)	C33—C34	1.383 (5)
C3—N1	1.425 (4)	C33—N3	1.421 (4)
C4—C5	1.393 (5)	C34—C35	1.387 (5)
C4—H4	0.9300	C34—H34	0.9300
C5—C6	1.391 (5)	C35—C36	1.390 (5)
C5—N2	1.417 (4)	C35—N4	1.427 (4)
C6—H6	0.9300	C36—H36	0.9300
C7—C8	1.390 (5)	C37—C38	1.387 (5)
C7—N1	1.398 (4)	C37—N3	1.398 (4)
C7—C12	1.406 (5)	C37—C42	1.403 (5)
C8—C9	1.377 (5)	C38—C39	1.382 (5)
C8—H8	0.9300	C38—H38	0.9300
C9—C10	1.384 (6)	C39—C40	1.387 (6)
C9—H9	0.9300	C39—H39	0.9300
C10—C11	1.378 (5)	C40—C41	1.375 (6)
C10—H10	0.9300	C40—H40	0.9300
C11—C12	1.397 (5)	C41—C42	1.401 (5)
C11—H11	0.9300	C41—H41	0.9300
C12—C13	1.444 (5)	C42—C43	1.445 (5)
C13—C14	1.387 (5)	C43—C44	1.389 (5)
C13—C18	1.411 (4)	C43—C48	1.398 (5)
C14—C15	1.386 (5)	C44—C45	1.360 (6)
C14—H14	0.9300	C44—H44	0.9300
C15—C16	1.390 (5)	C45—C46	1.388 (6)
C15—H15	0.9300	C45—H45	0.9300
C16—C17	1.380 (5)	C46—C47	1.380 (6)
C16—H16	0.9300	C46—H46	0.9300
C17—C18	1.394 (5)	C47—C48	1.385 (5)
C17—H17	0.9300	C47—H47	0.9300
C18—N1	1.390 (4)	C48—N3	1.400 (4)
C19—C20	1.380 (5)	C49—C50	1.382 (5)
C19—N2	1.393 (4)	C49—N4	1.394 (4)
C19—C24	1.413 (5)	C49—C54	1.405 (5)
C20—C21	1.356 (5)	C50—C51	1.385 (6)
C20—H20	0.9300	C50—H50	0.9300
C21—C22	1.387 (6)	C51—C52	1.387 (6)
C21—H21	0.9300	C51—H51	0.9300
C22—C23	1.377 (5)	C52—C53	1.372 (7)
C22—H22	0.9300	C52—H52	0.9300
C23—C24	1.383 (5)	C53—C54	1.393 (6)
C23—H23	0.9300	C53—H53	0.9300
C24—C25	1.445 (5)	C54—C55	1.449 (6)
C25—C26	1.397 (5)	C55—C56	1.388 (5)
C25—C30	1.403 (5)	C55—C60	1.398 (5)
C26—C27	1.369 (5)	C56—C57	1.365 (7)
C26—H26	0.9300	C56—H56	0.9300
C27—C28	1.385 (6)	C57—C58	1.395 (7)
C27—H27	0.9300	C57—H57	0.9300
C28—C29	1.382 (5)	C58—C59	1.385 (6)
C28—H28	0.9300	C58—H58	0.9300
C29—C30	1.384 (5)	C59—C60	1.385 (5)
C29—H29	0.9300	C59—H59	0.9300
C30—N2	1.407 (4)	C60—N4	1.399 (4)
			
C6—C1—C2	122.0 (3)	C34—C33—C32	120.8 (3)
C6—C1—I1	118.6 (2)	C34—C33—N3	119.8 (3)
C2—C1—I1	119.4 (3)	C32—C33—N3	119.5 (3)
C3—C2—C1	118.6 (3)	C33—C34—C35	119.7 (3)
C3—C2—H2	120.7	C33—C34—H34	120.1
C1—C2—H2	120.7	C35—C34—H34	120.1
C4—C3—C2	120.7 (3)	C34—C35—C36	120.4 (3)
C4—C3—N1	120.1 (3)	C34—C35—N4	119.8 (3)
C2—C3—N1	119.1 (3)	C36—C35—N4	119.8 (3)
C3—C4—C5	119.7 (3)	C31—C36—C35	118.7 (3)
C3—C4—H4	120.2	C31—C36—H36	120.7
C5—C4—H4	120.2	C35—C36—H36	120.7
C6—C5—C4	120.2 (3)	C38—C37—N3	129.4 (3)
C6—C5—N2	119.4 (3)	C38—C37—C42	122.3 (3)
C4—C5—N2	120.4 (3)	N3—C37—C42	108.3 (3)
C1—C6—C5	118.8 (3)	C39—C38—C37	117.4 (4)
C1—C6—H6	120.6	C39—C38—H38	121.3
C5—C6—H6	120.6	C37—C38—H38	121.3
C8—C7—N1	129.6 (3)	C38—C39—C40	121.3 (4)
C8—C7—C12	121.9 (3)	C38—C39—H39	119.4
N1—C7—C12	108.5 (3)	C40—C39—H39	119.4
C9—C8—C7	116.9 (4)	C41—C40—C39	121.4 (4)
C9—C8—H8	121.5	C41—C40—H40	119.3
C7—C8—H8	121.5	C39—C40—H40	119.3
C8—C9—C10	122.4 (4)	C40—C41—C42	118.8 (4)
C8—C9—H9	118.8	C40—C41—H41	120.6
C10—C9—H9	118.8	C42—C41—H41	120.6
C11—C10—C9	120.5 (4)	C41—C42—C37	118.8 (3)
C11—C10—H10	119.7	C41—C42—C43	133.5 (3)
C9—C10—H10	119.7	C37—C42—C43	107.6 (3)
C10—C11—C12	119.0 (3)	C44—C43—C48	119.6 (3)
C10—C11—H11	120.5	C44—C43—C42	133.8 (3)
C12—C11—H11	120.5	C48—C43—C42	106.6 (3)
C11—C12—C7	119.2 (3)	C45—C44—C43	119.4 (4)
C11—C12—C13	133.4 (3)	C45—C44—H44	120.3
C7—C12—C13	107.5 (3)	C43—C44—H44	120.3
C14—C13—C18	119.4 (3)	C44—C45—C46	120.5 (4)
C14—C13—C12	134.3 (3)	C44—C45—H45	119.7
C18—C13—C12	106.3 (3)	C46—C45—H45	119.7
C15—C14—C13	119.6 (3)	C47—C46—C45	121.8 (4)
C15—C14—H14	120.2	C47—C46—H46	119.1
C13—C14—H14	120.2	C45—C46—H46	119.1
C14—C15—C16	120.1 (3)	C46—C47—C48	117.2 (4)
C14—C15—H15	120.0	C46—C47—H47	121.4
C16—C15—H15	120.0	C48—C47—H47	121.4
C17—C16—C15	122.1 (3)	C47—C48—C43	121.4 (3)
C17—C16—H16	119.0	C47—C48—N3	129.5 (3)
C15—C16—H16	119.0	C43—C48—N3	109.0 (3)
C16—C17—C18	117.6 (3)	C50—C49—N4	129.6 (3)
C16—C17—H17	121.2	C50—C49—C54	121.7 (3)
C18—C17—H17	121.2	N4—C49—C54	108.7 (3)
N1—C18—C17	129.4 (3)	C49—C50—C51	117.9 (4)
N1—C18—C13	109.2 (3)	C49—C50—H50	121.1
C17—C18—C13	121.3 (3)	C51—C50—H50	121.1
C20—C19—N2	130.6 (3)	C50—C51—C52	121.2 (4)
C20—C19—C24	120.5 (3)	C50—C51—H51	119.4
N2—C19—C24	108.8 (3)	C52—C51—H51	119.4
C21—C20—C19	119.0 (4)	C53—C52—C51	120.7 (4)
C21—C20—H20	120.5	C53—C52—H52	119.7
C19—C20—H20	120.5	C51—C52—H52	119.7
C20—C21—C22	121.2 (4)	C52—C53—C54	119.7 (4)
C20—C21—H21	119.4	C52—C53—H53	120.2
C22—C21—H21	119.4	C54—C53—H53	120.2
C23—C22—C21	120.8 (4)	C53—C54—C49	118.8 (4)
C23—C22—H22	119.6	C53—C54—C55	134.2 (4)
C21—C22—H22	119.6	C49—C54—C55	106.8 (3)
C22—C23—C24	119.0 (4)	C56—C55—C60	119.6 (4)
C22—C23—H23	120.5	C56—C55—C54	133.3 (4)
C24—C23—H23	120.5	C60—C55—C54	107.1 (3)
C23—C24—C19	119.4 (3)	C57—C56—C55	118.7 (4)
C23—C24—C25	133.8 (3)	C57—C56—H56	120.6
C19—C24—C25	106.7 (3)	C55—C56—H56	120.6
C26—C25—C30	119.2 (3)	C56—C57—C58	121.5 (4)
C26—C25—C24	133.3 (3)	C56—C57—H57	119.2
C30—C25—C24	107.4 (3)	C58—C57—H57	119.2
C27—C26—C25	119.2 (3)	C59—C58—C57	120.7 (5)
C27—C26—H26	120.4	C59—C58—H58	119.6
C25—C26—H26	120.4	C57—C58—H58	119.6
C26—C27—C28	120.7 (4)	C60—C59—C58	117.4 (4)
C26—C27—H27	119.6	C60—C59—H59	121.3
C28—C27—H27	119.6	C58—C59—H59	121.3
C29—C28—C27	121.7 (4)	C59—C60—C55	122.0 (4)
C29—C28—H28	119.1	C59—C60—N4	129.3 (3)
C27—C28—H28	119.1	C55—C60—N4	108.7 (3)
C28—C29—C30	117.5 (3)	C18—N1—C7	108.5 (3)
C28—C29—H29	121.3	C18—N1—C3	125.8 (3)
C30—C29—H29	121.3	C7—N1—C3	125.2 (3)
C29—C30—C25	121.7 (3)	C19—N2—C30	108.5 (3)
C29—C30—N2	129.8 (3)	C19—N2—C5	125.7 (3)
C25—C30—N2	108.4 (3)	C30—N2—C5	125.7 (3)
C36—C31—C32	122.0 (3)	C37—N3—C48	108.4 (3)
C36—C31—I2	118.8 (3)	C37—N3—C33	125.9 (3)
C32—C31—I2	119.1 (2)	C48—N3—C33	125.7 (3)
C31—C32—C33	118.3 (3)	C49—N4—C60	108.7 (3)
C31—C32—H32	120.8	C49—N4—C35	126.5 (3)
C33—C32—H32	120.8	C60—N4—C35	124.9 (3)
			
C6—C1—C2—C3	1.7 (5)	C37—C42—C43—C44	−179.7 (4)
I1—C1—C2—C3	−177.2 (3)	C41—C42—C43—C48	−179.6 (4)
C1—C2—C3—C4	0.9 (5)	C37—C42—C43—C48	−0.3 (4)
C1—C2—C3—N1	178.9 (3)	C48—C43—C44—C45	−0.3 (5)
C2—C3—C4—C5	−1.9 (5)	C42—C43—C44—C45	179.1 (4)
N1—C3—C4—C5	−179.9 (3)	C43—C44—C45—C46	−0.1 (6)
C3—C4—C5—C6	0.3 (5)	C44—C45—C46—C47	0.0 (7)
C3—C4—C5—N2	179.9 (3)	C45—C46—C47—C48	0.6 (6)
C2—C1—C6—C5	−3.2 (5)	C46—C47—C48—C43	−1.0 (6)
I1—C1—C6—C5	175.7 (3)	C46—C47—C48—N3	−179.2 (4)
C4—C5—C6—C1	2.2 (5)	C44—C43—C48—C47	0.8 (5)
N2—C5—C6—C1	−177.4 (3)	C42—C43—C48—C47	−178.7 (3)
N1—C7—C8—C9	179.5 (4)	C44—C43—C48—N3	179.4 (3)
C12—C7—C8—C9	−0.1 (5)	C42—C43—C48—N3	−0.1 (4)
C7—C8—C9—C10	−0.1 (6)	N4—C49—C50—C51	177.4 (4)
C8—C9—C10—C11	−0.3 (7)	C54—C49—C50—C51	−0.8 (5)
C9—C10—C11—C12	0.8 (6)	C49—C50—C51—C52	0.1 (6)
C10—C11—C12—C7	−1.0 (5)	C50—C51—C52—C53	0.4 (7)
C10—C11—C12—C13	179.7 (4)	C51—C52—C53—C54	−0.1 (7)
C8—C7—C12—C11	0.7 (5)	C52—C53—C54—C49	−0.6 (6)
N1—C7—C12—C11	−179.1 (3)	C52—C53—C54—C55	−177.2 (4)
C8—C7—C12—C13	−179.8 (3)	C50—C49—C54—C53	1.0 (5)
N1—C7—C12—C13	0.5 (4)	N4—C49—C54—C53	−177.5 (3)
C11—C12—C13—C14	−2.6 (7)	C50—C49—C54—C55	178.6 (3)
C7—C12—C13—C14	178.0 (4)	N4—C49—C54—C55	0.0 (4)
C11—C12—C13—C18	178.4 (4)	C53—C54—C55—C56	−3.7 (7)
C7—C12—C13—C18	−1.1 (4)	C49—C54—C55—C56	179.3 (4)
C18—C13—C14—C15	−0.9 (5)	C53—C54—C55—C60	176.2 (4)
C12—C13—C14—C15	−179.9 (3)	C49—C54—C55—C60	−0.7 (4)
C13—C14—C15—C16	0.7 (5)	C60—C55—C56—C57	0.5 (6)
C14—C15—C16—C17	0.2 (6)	C54—C55—C56—C57	−179.5 (4)
C15—C16—C17—C18	−0.8 (6)	C55—C56—C57—C58	−1.1 (7)
C16—C17—C18—N1	178.4 (3)	C56—C57—C58—C59	1.5 (7)
C16—C17—C18—C13	0.6 (5)	C57—C58—C59—C60	−1.2 (6)
C14—C13—C18—N1	−178.0 (3)	C58—C59—C60—C55	0.6 (6)
C12—C13—C18—N1	1.3 (4)	C58—C59—C60—N4	178.9 (4)
C14—C13—C18—C17	0.2 (5)	C56—C55—C60—C59	−0.3 (5)
C12—C13—C18—C17	179.5 (3)	C54—C55—C60—C59	179.7 (3)
N2—C19—C20—C21	177.4 (4)	C56—C55—C60—N4	−178.9 (3)
C24—C19—C20—C21	−0.3 (5)	C54—C55—C60—N4	1.2 (4)
C19—C20—C21—C22	1.5 (6)	C17—C18—N1—C7	−179.0 (3)
C20—C21—C22—C23	−1.4 (6)	C13—C18—N1—C7	−1.0 (4)
C21—C22—C23—C24	0.0 (6)	C17—C18—N1—C3	9.2 (6)
C22—C23—C24—C19	1.2 (5)	C13—C18—N1—C3	−172.8 (3)
C22—C23—C24—C25	−176.4 (4)	C8—C7—N1—C18	−179.4 (4)
C20—C19—C24—C23	−1.1 (5)	C12—C7—N1—C18	0.3 (4)
N2—C19—C24—C23	−179.2 (3)	C8—C7—N1—C3	−7.5 (6)
C20—C19—C24—C25	177.1 (3)	C12—C7—N1—C3	172.2 (3)
N2—C19—C24—C25	−1.0 (4)	C4—C3—N1—C18	−121.8 (4)
C23—C24—C25—C26	−2.8 (7)	C2—C3—N1—C18	60.1 (5)
C19—C24—C25—C26	179.4 (4)	C4—C3—N1—C7	67.7 (5)
C23—C24—C25—C30	178.4 (4)	C2—C3—N1—C7	−110.4 (4)
C19—C24—C25—C30	0.6 (4)	C20—C19—N2—C30	−176.8 (4)
C30—C25—C26—C27	−0.6 (5)	C24—C19—N2—C30	1.1 (4)
C24—C25—C26—C27	−179.2 (4)	C20—C19—N2—C5	2.8 (6)
C25—C26—C27—C28	1.2 (6)	C24—C19—N2—C5	−179.3 (3)
C26—C27—C28—C29	−0.5 (6)	C29—C30—N2—C19	−178.5 (4)
C27—C28—C29—C30	−1.0 (6)	C25—C30—N2—C19	−0.7 (4)
C28—C29—C30—C25	1.6 (5)	C29—C30—N2—C5	1.9 (6)
C28—C29—C30—N2	179.2 (3)	C25—C30—N2—C5	179.7 (3)
C26—C25—C30—C29	−0.9 (5)	C6—C5—N2—C19	−132.6 (3)
C24—C25—C30—C29	178.1 (3)	C4—C5—N2—C19	47.8 (5)
C26—C25—C30—N2	−179.0 (3)	C6—C5—N2—C30	47.0 (5)
C24—C25—C30—N2	0.0 (4)	C4—C5—N2—C30	−132.6 (3)
C36—C31—C32—C33	0.3 (5)	C38—C37—N3—C48	178.0 (3)
I2—C31—C32—C33	177.0 (3)	C42—C37—N3—C48	−0.6 (4)
C31—C32—C33—C34	−1.3 (5)	C38—C37—N3—C33	−5.0 (6)
C31—C32—C33—N3	178.7 (3)	C42—C37—N3—C33	176.4 (3)
C32—C33—C34—C35	1.2 (5)	C47—C48—N3—C37	178.9 (4)
N3—C33—C34—C35	−178.7 (3)	C43—C48—N3—C37	0.5 (4)
C33—C34—C35—C36	−0.2 (5)	C47—C48—N3—C33	1.8 (6)
C33—C34—C35—N4	177.7 (3)	C43—C48—N3—C33	−176.5 (3)
C32—C31—C36—C35	0.7 (6)	C34—C33—N3—C37	127.3 (4)
I2—C31—C36—C35	−176.1 (3)	C32—C33—N3—C37	−52.6 (5)
C34—C35—C36—C31	−0.7 (5)	C34—C33—N3—C48	−56.2 (5)
N4—C35—C36—C31	−178.7 (3)	C32—C33—N3—C48	123.9 (4)
N3—C37—C38—C39	−178.9 (3)	C50—C49—N4—C60	−177.7 (4)
C42—C37—C38—C39	−0.5 (5)	C54—C49—N4—C60	0.7 (4)
C37—C38—C39—C40	−0.7 (6)	C50—C49—N4—C35	3.2 (6)
C38—C39—C40—C41	1.1 (7)	C54—C49—N4—C35	−178.4 (3)
C39—C40—C41—C42	−0.2 (6)	C59—C60—N4—C49	−179.6 (4)
C40—C41—C42—C37	−0.9 (5)	C55—C60—N4—C49	−1.2 (4)
C40—C41—C42—C43	178.4 (4)	C59—C60—N4—C35	−0.4 (6)
C38—C37—C42—C41	1.3 (5)	C55—C60—N4—C35	178.0 (3)
N3—C37—C42—C41	−180.0 (3)	C34—C35—N4—C49	59.1 (5)
C38—C37—C42—C43	−178.2 (3)	C36—C35—N4—C49	−123.0 (4)
N3—C37—C42—C43	0.6 (4)	C34—C35—N4—C60	−119.9 (4)
C41—C42—C43—C44	1.0 (7)	C36—C35—N4—C60	58.0 (5)
